# A Clinical Case of Anti-synthetase Syndrome: Polysymptomatic and Underdiagnosed

**DOI:** 10.7759/cureus.96690

**Published:** 2025-11-12

**Authors:** Nuno Oliveira, Francisco San Martin, Rosa Amorim

**Affiliations:** 1 Internal Medicine, Unidade Local de Saude Do Oeste - Unidade Caldas da Rainha, Caldas da Rainha, PRT; 2 Internal Medicine, Unidade Local de Saúde do Oeste - Unidade Caldas da Rainha, Caldas da Rainha, PRT

**Keywords:** anti-pl-7, antisynthetase syndrome, interstitial lung disease, raynaud's, rhabdomyolysis

## Abstract

Antisynthetase syndrome (ASSD) encompasses a group of autoimmune diseases associated with the formation of antibodies against aminoacyl-tRNA synthetases (ARS). The medical literature describes a constellation of symptoms, including myositis, arthritis, Raynaud's phenomenon, interstitial lung disease (ILD), fever, skin rash, or "mechanic's hands." We present a case of a 51-year-old man admitted with SAS with a consumptive clinical picture, rhabdomyolysis, ILD, and myositis. Positive anti-PL-7 and anti-nuclear antibodies (ANA) were detected. After diagnostic confirmation, the patient was treated with high doses of methylprednisolone and subsequently with cyclophosphamide, with a good response to immunosuppressive therapy. This clinical case is of great importance given the rarity of the pathology and the diagnostic difficulty of a disease with such a wide range of clinical manifestations.

## Introduction

Antisynthetase syndrome (ASSD) is an autoimmune disease characterized by multisystemic involvement, primarily interstitial lung disease (ILD), commonly associated with myositis, non-erosive arthralgia, Raynaud's phenomenon, "mechanic's hands," skin rash, xerostomia and xerophthalmia (Sicca syndrome) and constitutional symptoms, such as weight loss or fever, associated with the presence of anti-ATRS antibodies [1-4]. This is a rare disease affecting 2 to 3 women for every man, with an average age of onset between 43 and 60 years. This pathology always requires, regardless of the clinical presentation, the presence of anti-aminoacyl-tRNA synthetase (anti-ARS) antibodies. Aminoacyl-tRNA synthetases are cytoplasmic proteins that catalyze the binding of an amino acid to its specific tRNA. Eight types of antibodies have been described: Jo-1 or histidine-tRNA synthetase; PL-7 or threonine-tRNA synthetase; PL-12 or alanine-tRNA synthetase; OJ or isoleucine-tRNA synthetase; EJ or glycine-tRNA synthetase; KS or asparagine-tRNA synthetase; Zo or phenylalanine-tRNA synthetase; and Ha or tyrosine-tRNA synthetase [1,2]. According to scientific literature, different types of antibodies determine different clinical presentations, although there is always some clinical overlap between different antibodies and between this disease and others, such as idiopathic myopathies [3-5]. However, it should be noted that most patients will have ILD among the clinical manifestations listed.

Thus, the most frequent manifestations are anti-Jo1 antibodies, which, in addition to ILD, present with myositis, with 25-30% of patients with myositis testing positive for these antibodies, compared to the remaining antibodies, which reach values between 2% and 5% on average [1,2]. In addition to presenting myositis more frequently, patients with anti-Jo1 antibodies have more severe myositis, as well as more severe arthralgia and an increased risk of neoplasia [5,6]. Of the patients with anti-PL7 and anti-PL12 antibodies, those with anti-Jo1 antibodies develop an earlier and more severe form of ILD [5,7-11].

However, other symptoms may be associated with anti-ARS antibodies, resulting in confounding factors. Given that, the definition of ASSD includes the aforementioned list of symptoms associated with positive anti-ARS antibodies. This does not exclude the possibility of additional symptoms. Thus, patients with anti-PL7 and anti-PL12 often have gastrointestinal symptoms and may also be associated with pericarditis and necrotizing myopathy, respectively [2-6,12,13].

## Case presentation

The patient was a 51-year-old male from Nepal. He had been living in Portugal for two years. Professionally, he worked in the restaurant industry for 10 years. He lived at home with his wife and son. He did not speak Portuguese and spoke English with difficulty. There was no recent history of domestic or international travel, pets, known toxicophilia, smoking, or risky behaviors.

**Figure 1:**
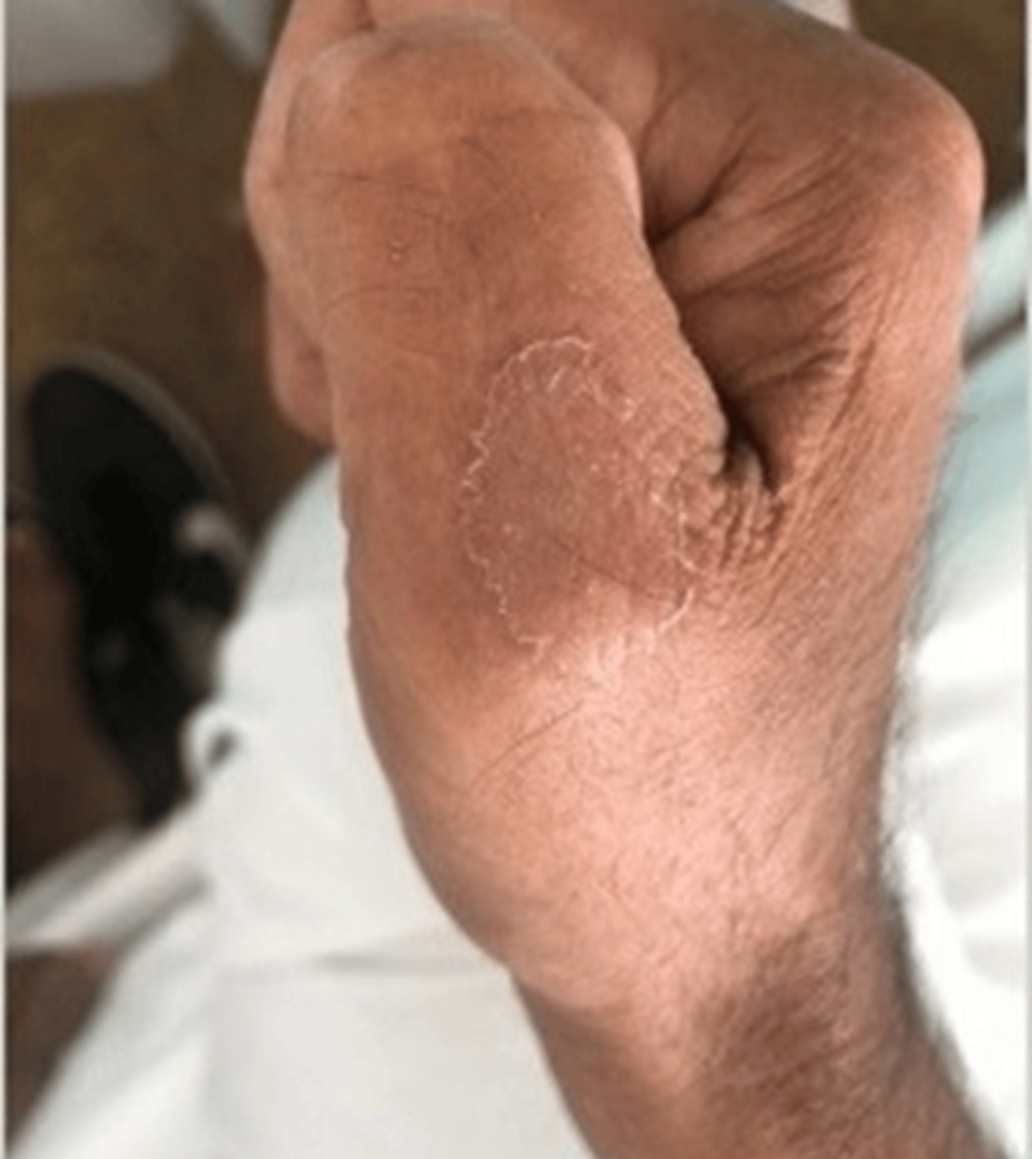
Sclerotic lesion on the dorsal part of the first finger

**Figure 2:**
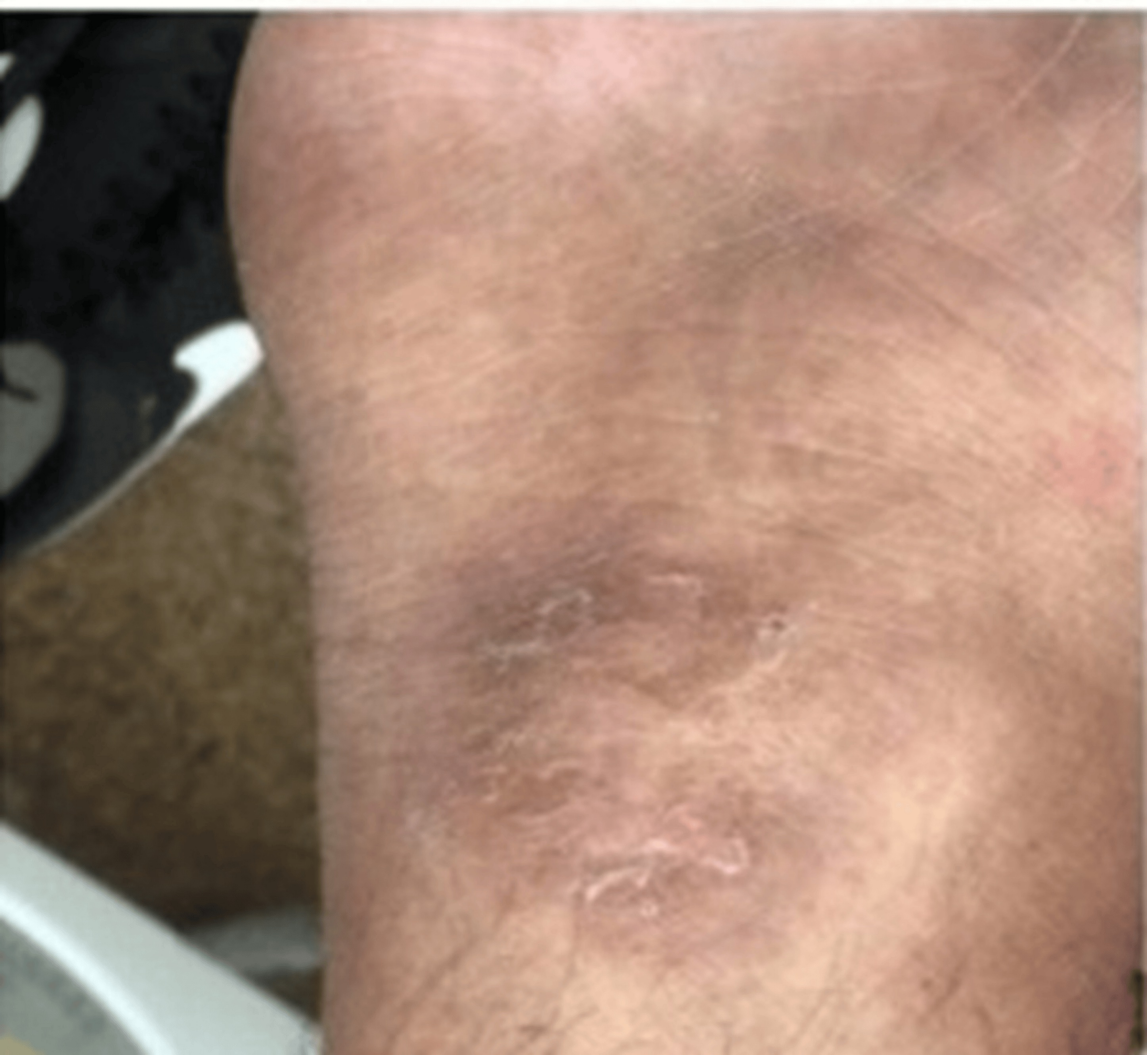
Sclerotic lesion on the forearm

**Figure 3:**
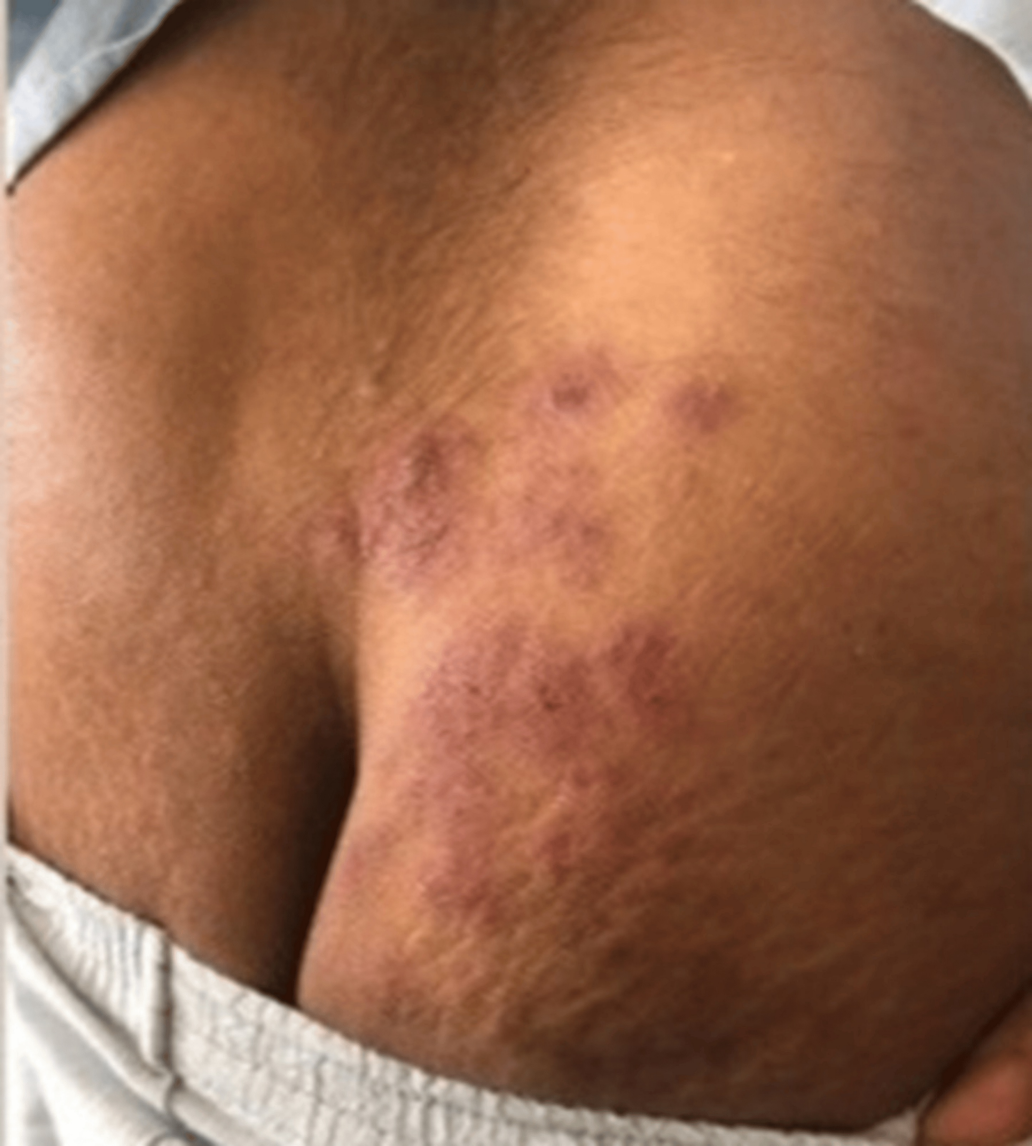
Sclerotic lesions on the right buttock

**Figure 4:**
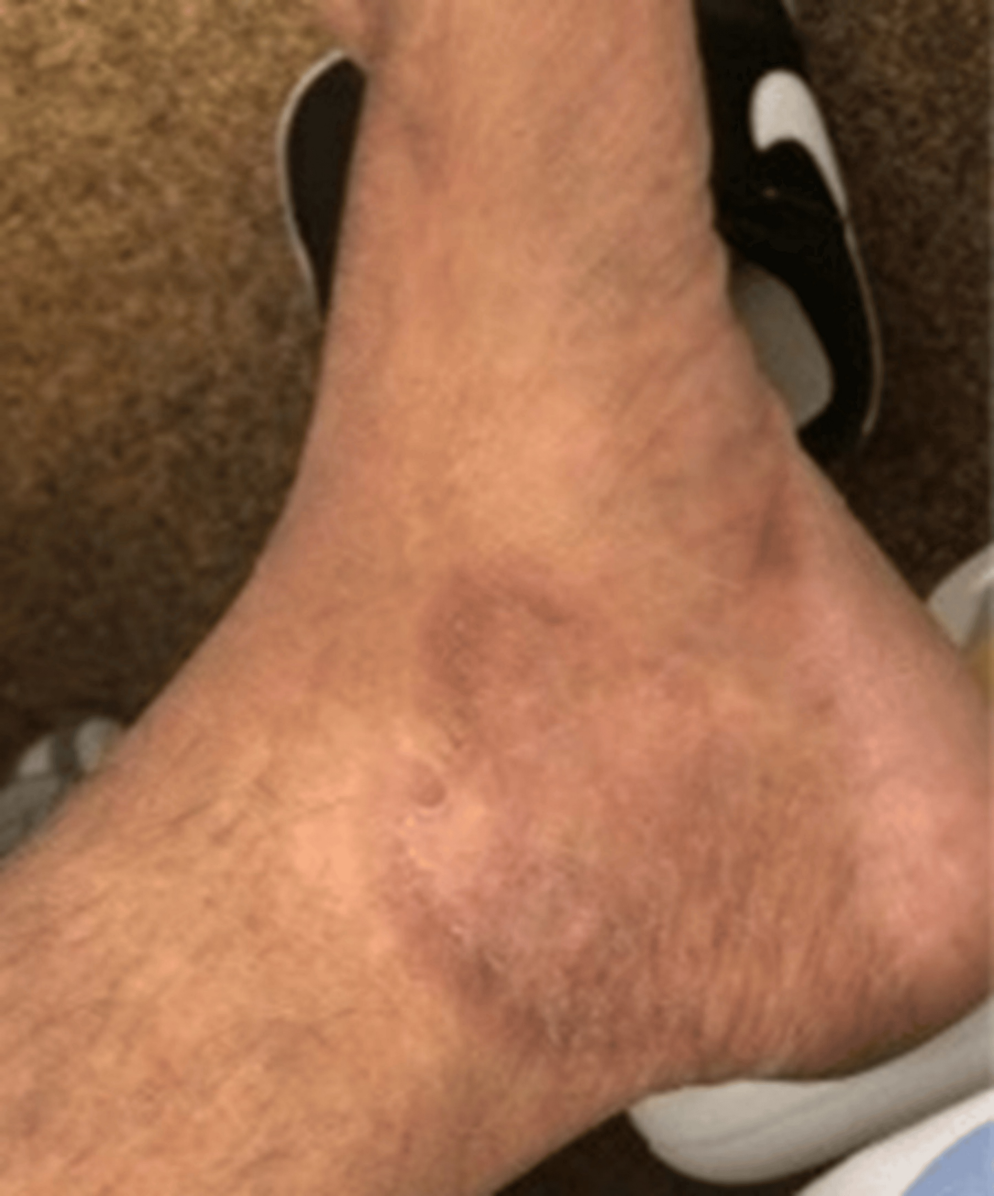
Sclerotic lesions on the left foot

**Figure 5:**
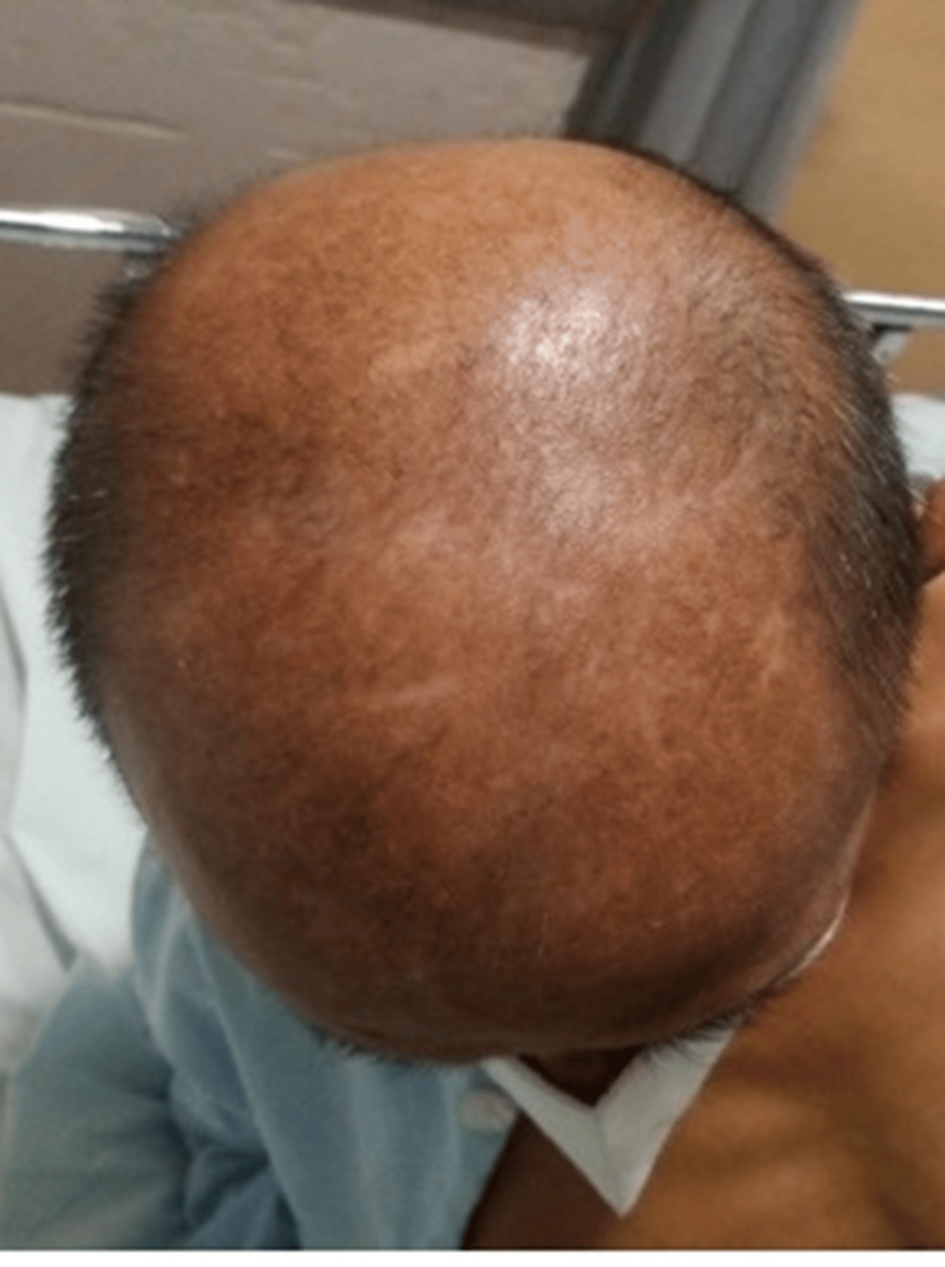
Sclerotic lesions on the top of the head

The patient reported the onset, approximately 7 months ago, of asthenia/adynamia associated with weight loss (according to the patient he lost approximately 38.5 kg), anorexia, dyspnea (no predominant time of day, initially progressive, but stable in recent months), dysphagia (intermittent, mainly associated with solids), localized skin sclerosis with well-defined margins (on the right hand, groin area, scalp, and left ankle), as shown in Figures 1-5.

He also reported nocturnal hyperhidrosis, generalized myalgia (with progressive muscle weakness and restriction of daily activities), and symmetrical arthralgia in the knee, shoulder, and elbow joints. In addition, color change of the hands, especially the fingers, has been described, occurring both during work and when walking outdoors, accompanied by cyanosis. The patient also reports similar symptoms in the lower limbs, although less frequently.

Since the onset of the illness, he has made several visits to the emergency department (a total of five) with similar complaints. In April 2021, he was seen in the emergency department, where he was diagnosed with an infectious exacerbation of pulmonary fibrosis, and he was discharged with a prescription for amoxicillin-clavulanic acid and azithromycin. He returned on August 31, 2021, due to clinical worsening and was admitted to the Internal Medicine Service of Peniche.

**Figure 6:**
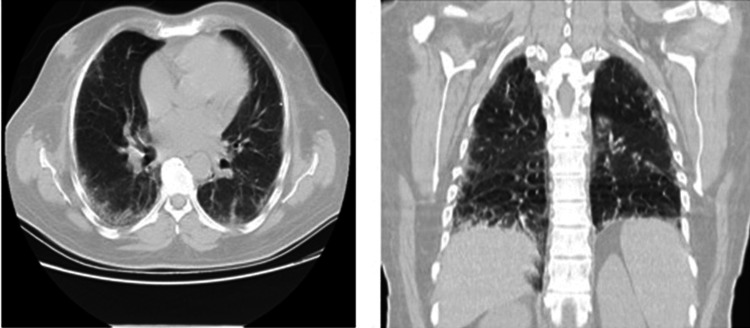
Chest CT scan left: horizontal section; right: frontal section

During hospitalization, a chest CT scan, shown in Figure 6, identified areas with ground-glass opacity and scattered linear and subpleural densifying lesions in the periphery of the bilateral lung parenchyma, more evident in the right basal lobe, without atypical viral features, to be clinically correlated. Multiple small mediastinal and hilar adenopathies were also revealed.

The diagnosis was interstitial pneumonia or superinfected pulmonary fibrosis, and empirical antibiotic therapy with levofloxacin was administered. An abdominal-pelvic CT scan was also performed, showing inguinal adenopathies, with no other significant changes. On September 15, a bronchofibroscopy was performed, which showed no observable macroscopic changes. In this study, samples were collected for cytomorphological, bacteriological, mycobacteriological, and PCR testing for *Mycobacterium tuberculosis* (interferon gamma release assay (IGRA) testing was also requested, which came back negative). The study also included neoplastic cell testing. The samples ruled out the clinical hypothesis of tuberculosis, neoplastic cells, or other significant changes. Thus, an analytical study of other infectious or autoimmune causes, or possibly paraneoplastic causes that could justify the constitutional picture, was also performed. Analytically, the patient had no leukocytosis or elevated C-reactive protein (CRP); the serum protein electrophoresis had no monoclonal peaks, with an erythrocyte sedimentation rate (ESR) of 83 mm/h and a consistent increase in creatine kinase between 3283 and 4230; serology for Cytomegalovirus (CMV), Epstein-Barr virus (EBV), hepatitis, syphilis, and mononucleosis was negative; IGRA was negative; aldolase was 103.5; angiotensin converting enzyme (ACE) was normal. Autoimmunity markers, antinuclear antibody (ANA), anti-double-stranded DNA (ds-DNA) antibodies, anticytrulline antibodies, antihistone antibodies, antinucleosome antibodies, anti-nuclear and cytoplasmic antibodies, anti-Jo-1, and anti-nuclear and cytoplasmic antibodies (anti-SSA) were also requested, which were negative. Other autoimmune tests were also requested, the results of which were not available at the time of the patient's discharge, namely, anti-mitochondrial antibody (AMA), anti-smooth muscle antibodies (SMA), anti-liver kidney microsomal antibody type 1 (anti-LKM1), and antibodies for myositis.

**Figure 7:**
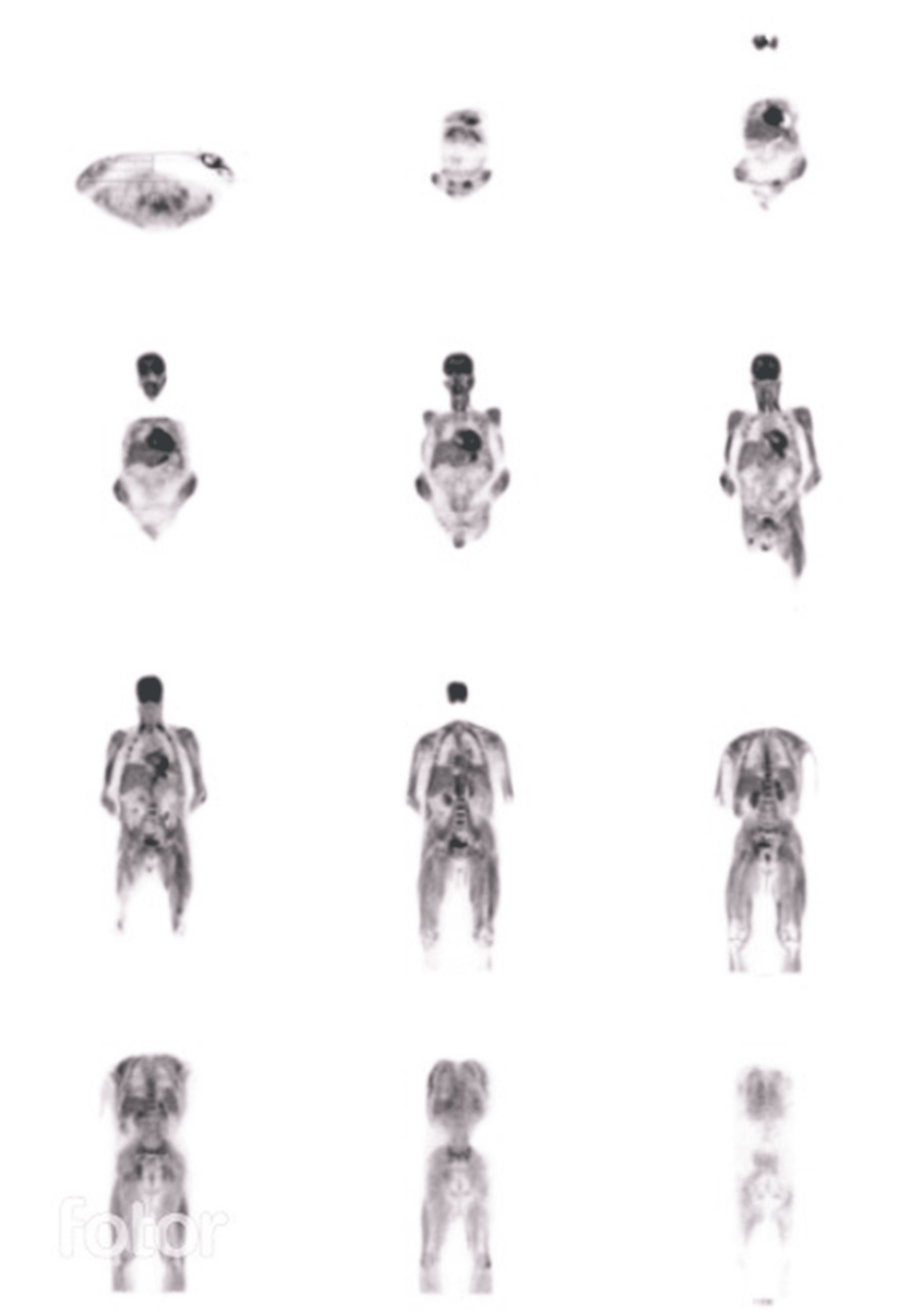
PET scan showing increased muscle uptake PET: positron emission tomography

The patient was therefore discharged on 17/9/2021 with an appointment for a follow-up consultation with Internal Medicine, pending some autoimmunity results and the positron emission tomography (PET) scan. At the consultation, the PET scan results were analyzed and showed intense muscle uptake (Figure 7).

The results of the remaining autoimmune tests were also known, with positive ANA and PL-7 myositis.

In this context, a diagnosis of autoimmune myositis with anti-synthetase syndrome was made. From conversations with the patient and his family since his discharge, a marked clinical worsening was noted, with continuous weight loss and complaints of electric shock-like pain in the right side of the body. Once again, an urgent consultation with an autoimmune specialist was requested. After discussion with the colleague, the patient was admitted and treatment was started with methylprednisolone in pulses of 1000 mg daily for 3 days, followed by immunosuppressive therapy with cyclophosphamide.

The patient tolerated the treatment with good clinical and laboratory progress. He was discharged on October 13 and referred to the autoimmune department of our hospital center.

## Discussion

The patient was admitted for investigation of a consumptive condition, and therefore, we initially considered three main hypotheses or diagnostic groups. We considered the possibility of a neoplastic disease, an autoimmune disease, or an infectious disease [7,10,14-16].

In the context of infectious diseases and given the visible fibrotic changes in the chest CT scan, a bronchoscopy was performed, which did not reveal any macroscopic changes, nor did any of the samples taken reveal any changes, namely, in the search for mycobacteria, as tuberculosis would be a strong possibility. Diseases such as EBV, CMV, and hepatitis were also ruled out serologically. The patient never had a significant elevation of inflammatory parameters, with the most pronounced value being the sedimentation rate, at around 83 mm/h. Although high, this value is nonspecific and continues to support the three hypotheses. On the other hand, muscle pain could be explained by an infectious agent, but constantly high, non-fluctuating rhabdomyolysis does not fit in with an infectious agent, whose infectivity varies over time, first in a phase of exponential growth, then in a stationary phase, and finally in a descending phase.

In terms of neoplasms, there were no imaging changes suggestive of a primary tumor site. Detectable, deep, non-palpable adenomegaly was noted, which may be reactive to infection or a manifestation of neoplasia. Since admission, the patient had presented with B symptoms, namely, marked weight loss, marked anorexia, and persistent fatigue or tiredness. However, neither imaging nor hematological tests revealed any significant changes, and there were no specific organ symptoms. In addition, scattered trophic skin changes, without inflammatory features and without apparent characteristics of malignancy, favored the third hypothesis.

The third hypothesis was that of an autoimmune disease. In this case, we would have a disease affecting the muscles and skin, accompanied by anorexia and weight loss, but without other particularly specific symptoms. We began investigating autoimmunity. The results of this type of investigation are generally slow and depend on the submission of samples to laboratories outside our hospital center. The autoimmunity test performed at our hospital center came back negative. The latest autoimmunity results only arrived after the PET results. These results with anti-synthetase antibodies validated the PET report, which was characterized by global muscle uptake and therefore justified the continuously elevated rhabdomyolysis, even at rest, without trauma and in a controlled hospital environment. The patient had PL7 positive and anti-Jo1 negative antibodies, meaning that, according to the literature, we were expecting a clinical picture with more intense ILD and less myalgias and perhaps the presence of gastrointestinal symptoms [5,7-9]. In our case, the myalgias were the dominant symptom, and although the initial reason for commitment was respiratory distress, the dyspnea was easily controlled. Additionally, there were no gastrointestinal symptoms and no pericarditis [2-5]. 

Nevertheless, the criteria for ASSD were fulfilled due to the presence of ILD, myositis, arthralgia, skin sclerosis symptoms, and significant weight loss in the presence of anti-ATRS antibodies [1-4].

Lastly, in terms of prognosis, there also appear to be differences according to positive anti-ARS antibodies. In a comparative study conducted by Aggarwal et al., the cumulative survival of anti-Jo1 was compared with that of other anti-ARS at 5 and 10 years. The survival rate of patients with anti-Jo1 was 90% and 70%, respectively, while for non-anti-Jo1 patients, it was 75% and 47% (p<0.005) [4,6,10-12]. The authors postulate that the difference is due to diagnostic difficulties/delays in the non-anti-Jo1 group and not to the pathology itself, since the same study indicates that the median time to diagnosis was 0.4 months (in the anti-Jo1 group) and 1 year (in the non-anti-Jo1 group) (p<0.001) [1,2,4,5,13].

## Conclusions

In summary, the number of possible diagnostic hypotheses for a consumptive syndrome is very extensive. For this reason, the patient was admitted, and a systematic diagnostic approach was taken. During hospitalization, the order in which we have our complementary resources available and their results is not always ideal; these are the limitations of a small hospital setting. Therefore, at any given time, there are always several clinical hypotheses and several tests underway, which inevitably leads to increased expenses, at the risk of delaying the diagnosis.

This case also highlights the importance of primary health care services. It would have been possible to make a more timely diagnosis, perhaps without the need for hospitalization, but two situations stand out: on the one hand, he was a Nepalese citizen not registered at any health center, so his only means of assistance was the emergency service, where, given the influx of patients, we may have had the opportunity to look at a situation like this and have the discernment to make the right choice; on the other hand, given the pandemic, there was also a fear among users of resorting to health services, which again leads to a delay in diagnosis and a progressively more serious clinical condition. This case serves as a reminder that in patients with new-onset idiopathic pulmonary fibrosis, with no apparent risk factors and especially when associated with rhabdomyolysis in patients with no triggering or justifying etiology, we should not fail to include the clinical hypothesis of antisynthetase syndrome in our range of diagnostic possibilities.
